# Improving Neuroplasticity through Robotic Verticalization Training in Patients with Minimally Conscious State: A Retrospective Study

**DOI:** 10.3390/brainsci14040319

**Published:** 2024-03-27

**Authors:** Rosaria De Luca, Antonio Gangemi, Mirjam Bonanno, Rosa Angela Fabio, Davide Cardile, Maria Grazia Maggio, Carmela Rifici, Giuliana Vermiglio, Daniela Di Ciuccio, Angela Messina, Angelo Quartarone, Rocco Salvatore Calabrò

**Affiliations:** 1 IRCCS Centro Neurolesi Bonino-Pulejo, Cda Casazza, SS 113, 98124 Messina, Italy; rosaria.deluca@irccsme.it (R.D.L.); antonio.gangemi@irccsme.it (A.G.); davide.cardile@irccsme.it (D.C.); mariagrazia.maggio@irccsme.it (M.G.M.); carmela.rifici@irccsme.it (C.R.); giuliana.vermiglio@irccsme.it (G.V.); daniela.diciuccio@irccsme.it (D.D.C.); angela.messina@irccsme.it (A.M.); angelo.quartarone@irccsme.it (A.Q.); roccos.calabro@irccsme.it (R.S.C.); 2 Department of Economics, University of Messina, 98100 Messina, Italy; rafabio@unime.it

**Keywords:** Erigo, robotic verticalization, neurorehabilitation, minimally conscious state, neuroplasticity, EEG analysis processing

## Abstract

In disorders of consciousness, verticalization is considered an effective type of treatment to improve motor and cognitive recovery. Our purpose is to investigate neurophysiological effects of robotic verticalization training (RVT) in patients with minimally conscious state (MCS). Thirty subjects affected by MCS due to traumatic or vascular brain injury, attending the intensive Neurorehabilitation Unit of the IRCCS Neurolesi (Messina, Italy), were included in this retrospective study. They were equally divided into two groups: the control group (CG) received traditional verticalization with a static bed and the experimental group (EG) received advanced robotic verticalization using the Erigo device. Each patient was evaluated using both clinical scales, including Levels of Cognitive Functioning (LCF) and Functional Independence Measure (FIM), and quantitative EEG pre (T0) and post each treatment (T1). The treatment lasted for eight consecutive weeks, and sessions were held three times a week, in addition to standard neurorehabilitation. In addition to a notable improvement in clinical parameters, such as functional (FIM) (*p* < 0.01) and cognitive (LCF) (*p* < 0.01) outcomes, our findings showed a significant modification in alpha and beta bands post-intervention, underscoring the promising effect of the Erigo device to influence neural plasticity and indicating a noteworthy difference between pre-post intervention. This was not observed in the CG. The observed changes in alpha and beta bands underscore the potential of the Erigo device to induce neural plasticity. The device’s custom features and programming, tailored to individual patient needs, may contribute to its unique impact on brain responses.

## 1. Introduction

Minimally conscious state (MCS) is a serious medical condition caused by damage to the brain due to vascular and/or traumatic events. It is characterized by clear, reproducible but inconsistent signs of consciousness of self or the surroundings [1]. The diagnosis of MCS is based primarily on clinical findings, including the ability to fixate or tracking visual stimuli, to follow simple commands, to manipulate, reach for or hold objects, verbal or gestural yes/no responses, and other response to specific environmental events that cannot be explained by simple reflexive activity [2]. Bruno and colleagues recently proposed a further subcategorization of MCS based on the complexity of observed behavioral responses, dividing it into minimally conscious state plus/minus [3].

MCS is generally distinguished from vegetative state (VS), which has recently been replaced by the term “unresponsive wakefulness syndrome” (UWS) [4]. In contrast to MCS, VS/UWS lacks interaction with the environment and other people as well as self-awareness. Both MCS and UWS are classified as chronic disorders of consciousness (DoC). These invaliding conditions may have other severe cognitive and motor-sensory impairments, including aphasia, agnosia, limb paralysis, spasticity, sensory disturbances, and cardiorespiratory complications, which can lead to a bedridden state [5,6]. One of the main goals of neurorehabilitation in this patient group is to prevent secondary medical complications while stimulating the patient by promoting arousal [7,8]. The primary aims of the current European Academy of Neurology (EAN) guidelines encompass two key aspects: firstly, to provide comprehensive insights into the diagnosis of DoC by integrating current evidence from three interrelated sources—namely bedside examination, functional neuroimaging and electroencephalography (EEG)—and secondly, to provide relevant clinical guidelines aimed at improving the efficiency of diagnosis, prognosis and clinical management strategies for individuals affected by such disorders [9]. However, while commendable, the guidelines pose several questions, including some related to the practical implementation of their recommendations.

In contrast to the European guidelines, the American guidelines pay particular attention to the needs of patients and their families and advocate comprehensive education and counseling initiatives aimed at explaining the diagnosis, prognosis, and treatment modalities to those affected [10]. There is a consensus in the medical community that a multimodal approach to the assessment of residual consciousness is imperative to advance the diagnosis of DoC. However, conducting such an assessment is a huge challenge in contrast to the relatively straightforward implementation of multidisciplinary rehabilitative interventions.

The implementation of recommendations outlined in European and American guidelines on DoC would benefit from further consideration of the concept of responsibility and the utilization of the Distributed Responsibility Model across interpersonal assessment to multimodal rehabilitative approaches. This model integrates various conventional and advanced rehabilitative methodologies [11].

In this context, among the non-pharmacological therapies, early intensive neurorehabilitation combining physical, occupational, speech, and neuropsychological therapy appears to improve long-term functional recovery [12,13,14,15,16]. Specialized rehabilitation protocols for DoC individuals are essential for improving recovery and long-term care. Moreover, in the last ten years, the use of innovative approaches (e.g., robotics and virtual reality) to further promote recovery has been implemented [17]. Among the robotic treatments, early verticalization with the Erigo device (Hocoma, Volketswil, Switzerland) is becoming of benefit, thanks to the lower limb movement and orthostatic position [18]. Indeed, the tool combines gradual verticalization, repetitive leg movement (which unites muscular functional electrical stimulation (FES) of the lower limbs with synchronized stepping exercises), and safe stabilization to improve body weight loading during the standing position. Robot-based neurorehabilitation is effective in enhancing brain plasticity, motor performance, and cognitive recovery in general. However, whether the Erigo can lead to improvements in brainwaves and neuroplasticity in patients with MCS is still an open question.

In fact, Rosenfelder et al. [19], by investigating the effects of robotic verticalization on Doc, have measured the means of high-density quantitative EEG at baseline, directly after the verticalization program and after 6 months, without reporting significant results. In this study, our main aim was to assess the impact of the Erigo device on variations in band power concerning brainwave parameters (theta, alpha, and beta) in both the left and right hemispheres by comparing pre-intervention baseline measurements (T0) with post-test results (T1). To obtain a differentiated assessment of the functionality of the cerebral hemispheres and their potential contribution to different cognitive and motor functions, we performed an analysis of the results of the band power spectra on the individual hemispheres separately after the power spectral density (PSD) calculated for each sensor. This allowed for a more detailed evaluation of the effects of therapy on individual cerebral hemispheres, which could provide a more comprehensive understanding of the effects of therapy on cognitive and motor function. The second aim involved a comprehensive clinical evaluation of the impact of RVT with Erigo on global cognitive and motor functioning.

## 2. Materials and Methods

### 2.1. Study Design and Population

Thirty patients affected by MCS (with a mean age of 57.73 ± 9.63) due to vascular or traumatic brain injury (at least 6 months after the event) attending the intensive Neurorehabilitation Unit of the IRCCS Centro Neurolesi “Bonino-Pulejo” (Messina, Italy), from December 2022 to September 2023, were analyzed using an electronic recovery system data.

Given the retrospective nature of our investigation, we used an electronic medical database to extract data. We used both cognitive and motor parameters to properly select the appropriate patients who had been treated with the Erigo device. The use of this robotic verticalization device is part of the standard care of some patients attending our rehabilitation unit. The collected clinical evaluations were performed before (T0) and after (T1) the training by rehabilitation team practitioners, which includes neurologists, physiatrists, nurses, physiotherapists, and psychologists.

We considered as inclusion criteria: (i) an age between 18 and 70 years; (ii) diagnosis of post-acute MCS (i.e., at least three months after the neurological event), due to vascular or traumatic events, according to the Simplified Evaluation of CONsciousness Disorders (SECONDs), which was administered at enrollment; (iii) adequate pulmonary gas exchanging function (arterial O_2_ pressure/O_2_ flux ratio 250); (iv) stable hemodynamics (absence of dangerous variations of Mean Arterial Pressure or Heart Rate). The exclusion criteria comprised several factors: (i) administration of sedatives; (ii) unstable intracranial pressure (ICP) values; (iii) cerebral perfusion pressure (CPP) below 60 mmHg; (iv) presence of skin lesions or fractures on the thorax, lower limbs, or abdomen; (v) occurrence of deep vein thrombosis; (vi) concurrent medical conditions with the potential to interfere with the process of verticalization; (vii) contraindications to the utilization of technological instrumentation, encompassing body weight exceeding 135 kg, leg length falling below 70 cm or surpassing 102 cm, pronounced muscle contractions at the hip, knee, and ankle, and lack of patient compliance.

All experiments were conducted according to the ethical procedures and policies approved by the local Ethics Committee (IRCCS-ME 19/22). All patients’ legal guardians gave their written informed consent to participate in the study and data publication. Consent was also requested of caregivers to publish the study results.

### 2.2. Procedures

The included MCS patients were equally divided into two groups, having the same demographic and medical characteristics but different rehabilitation treatments. They received either verticalization training (VT), with Erigo (RVT) for EG or without (VT) for CG, in addition to a conventional physiotherapy program. Both treatments (RVT and VT) were performed in a dedicated space, 3 times a week, lasting for 45 min per session, for about 8 weeks with a total of 24 sessions, as per our standard training protocol. Each patient was evaluated through clinical scales at the beginning (T0) and at the end (T1) of the study, by a clinician not involved in the study. In addition, a neurophysiology technician measured brain electrical activity through an electroencephalogram (EEG).

### 2.3. Verticalization Training (VT)

In the CG, MCS patients received 45 min of training for each session, consisting of postural change training (e.g., from supine to sitting; from to sitting to standing position). Postural changes were achieved through preparatory exercise, following the Bobath method, which is the most common physiotherapy technique used in neurorehabilitation. The sitting posture was reached in bed through a half side rail, which is a safety device commonly used in beds, or bed trapeze, consisting of a mobility aid installed above or next to a bed. These tools were used to assist patients with adjusting their position in the bed, promoting turning, lifting, and transferring from the bed to the wheelchair.

Since hemiplegic patients tended to lean toward the affected side while seated, balance training involved overcorrection towards the unaffected side. Once the patient could sit up in bed, they were encouraged to move to the bedside. Subsequently, procedures for standing and transferring from bed to chair (and vice versa) could commence. Upon successfully completing these stages, the patient progressed to VT using the same paradigm as RVT, facilitated by a simple tilt table. Moreover, during this training, static balancing exercises requiring body weight shifting from one leg to the other, to make the patient able to sustain themselves during the standing position.

### 2.4. Robotic Verticalization Training (RVT)

**Figure 1:**
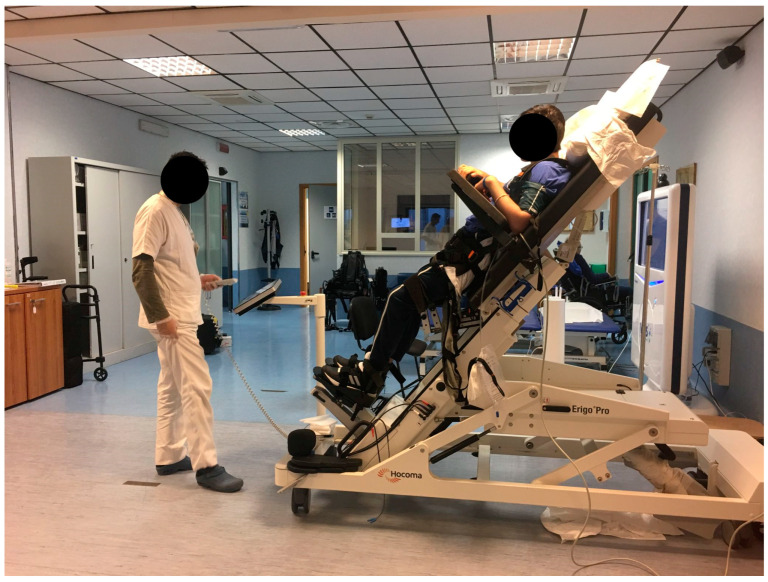
Shows an RVT session in a patient with MCS using Erigo device.

Erigo is a tilt table equipped with electronically controlled leg movement mechanism. It allows gradual verticalization and facilitates the mobilization of in-patients, preventing secondary bedridden complications, due to prolonged immobility (Figure 1).

Erigo promotes gradual robotic verticalization, in addition to passive leg movement and alternating loading and unloading of the patient’s legs, according to the patients’ needs. During the first session of about 25 min, efforts were made to adapt the patient to the device. Thereafter, the session time increased to 45 min, and the tilt of the table gradually increased (from 45° to 90°), as well as the speed of stepping exercises calibrated depending on the patient’s clinical conditions and needs. Moreover, cycle leg movement provided by Erigo can be personalized.

### 2.5. Conventional Physiotherapy Program

Both groups received conventional physiotherapy program, in addition to verticalization training with or without Erigo device, which included passive and assisted joint mobilization, muscle stretching in upper and lower limbs, breathing control exercises, proprioceptive exercises with various surfaces of different consistence, postural and trunk stabilization in sitting position. This conventional physiotherapy program was performed for 45 min, 6 days a week for the duration of hospitalization [19,20,21,22,23,24].

### 2.6. Clinical Outcomes

All MCS patients were clinically evaluated before (T0) and after (T1) our protocol. In particular, a physician administered the Levels of Cognitive Functioning scale (LCF) [25], which is used to evaluate the cognitive performances in post-coma patients, and the Functional Independence Measure (FIM), a tool consisting in 18-item (13 motor (motFIM) and 5 cognitive (cognFIM)) that make it possible to explore social, psychological, and physical function, used to assess the patient’s level of dependence in daily life activities [26]. Additionally, only at T0, all patients were evaluated with the Simplified Evaluation of CONsciousness Disorders (SECONDs), a fast and valid instrument that includes eight items that help to determine consciousness level in patients with brain injury. The items contained in the test cover: observation, command-following, communication (intentional or functional—conditional item), visual pursuit, visual fixation, localization to pain (conditional item), oriented behaviors, and arousal [27].

### 2.7. Electroencephalographic Measurements

During EEG recording procedure, meticulous measures were undertaken to maintain a quiet environment, devoid of extraneous stimuli, to mitigate external influences. The patient was comfortably situated either in a chair or on a bed, contingent upon individual preference and comfort requirements. Illumination levels were moderated or adjusted to foster a serene setting conducive to relaxation. Provision of a light cover or eye mask was made available as needed to attenuate ambient luminosity, thereby minimizing potential perturbation of EEG signals. The neurophysiological technician and attending medical personnel executed procedures with precision to minimize inadvertent movements and disturbances during the recording session, thus safeguarding data integrity. A sophisticated digital EEG amplifier, specifically the Micromed Medical System (Treviso, Italy), was employed to capture EEG signals. A notch philter was applied at 50 Hz to reduce interference from power lines. Visual inspection was used to manually remove artifacts. The data were then segmented into artifact-free epochs, each lasting 4 s.

In investigating changes in neuroplasticity, our study focused on the power spectrum bands comprising the theta, alpha, and beta EEG rhythms. These different frequency bands of brain electrical activity are closely linked to various cognitive functions and neuronal plasticity phenomena. The main objective was to evaluate the individual effects of robotic rehabilitation on brain plasticity in individuals diagnosed with MCS. EEG data acquisition was performed using a conventional digital EEG amplifier from Micromed Medical System, (Treviso, Italy). This system allowed continuous recording of EEG signals on 19 channels, with electrode placement following the criteria of the International 10/20 System, where the reference electrode was positioned at the mastoids (A1) and the ground electrode posteriorly at Fz.

After EEG data acquisition, preprocessing was performed using MATLAB (version 7.10.0, MathWorks Inc., Natick, MA, USA, 2010) in conjunction with EEGLAB [28]. First, baseline normalization was performed for each channel, followed by high-pass filtering at 0.5 Hz to attenuate respiratory artifacts and low-pass filtering at a cut-off frequency of 50 Hz to suppress high-frequency noise. To analyze the brain rhythms, the EEG data from the occipital regions were examined for the alpha rhythm, while the data from the frontotemporal regions were examined bilaterally for beta and theta rhythms. Electrode placement spanned the entire scalp to ensure comprehensive coverage. EEG recordings were performed during a 20-min interval in which patients were in a state of psycho-sensory rest with eyes closed, interspersed with brief intervals in which the eyes were opened to assess alpha rhythm reactivity in the occipital cortex areas. Quantitative analysis was performed using customized algorithms developed in the MATLAB environment (version 7.10.0, MathWorks Inc., Natick, MA, USA, 2010). The power spectral density (PSD) calculations were performed using the Welch method to transform the signals from the time domain to the frequency domain. The PSD values were calculated for each epoch and then averaged. Both the absolute total power and the power of the individual bands were calculated for each electrode, taking into account the frequency bands theta (4–7 Hz), alpha (8–13 Hz), and beta (14–29 Hz).

#### Statistical Analysis

Data analysis was conducted using IBM SPSS Statistics, Version 24 (IBM Corp., Armonk, NY, USA). The significance level for statistical tests was set at *p* < 0.05. For the analysis of neurophysiological measures, we employed a MANOVA model for repeated measures with a between-subject factor (group: experimental and control) and two within-subject factors: hemisphere side (left and right) and phases (T0—pre-intervention baseline, T1—post-test) for each band power (theta, alpha, and beta). For clinical measures, we utilized two ANOVA models for repeated measures with a between-subject factor (group: experimental and control) and a within-subject factor (phases: T0—pre-intervention baseline and T1—post-test). Contrast analysis was applied to further examine the nature of the significant interaction and provide a more nuanced understanding of group differences over time. In case of significant effects, the effect size of the test was reported, computed, and categorized according to eta squared η^2^.

Furthermore, to provide a comprehensive assessment, we applied paired *t*-tests within each group (CG T0–T1 and EG T0–T1) and independent *t*-tests between the two groups (CG vs. EG at T0 and at T1) for clinical outcome measures. We assessed the assumption of normality using the Shapiro–Wilk test and examined the homogeneity of group variances using Levene’s test. The data analyzed had a normal distribution, the test was not significant, so Student *t*-tests, using the Bonferroni correction, were used for post-hoc testing of group differences in time and performance. Power analysis using Cohen’s d as the effect size parameter was applied.

## 3. Results

Training was completed in all the subjects involved without any adverse events. No significant differences emerged at baseline (T0) between the two groups concerning demographic variables, etiology, and psychometric/outcome scores (see Table 1).

### 3.1. EEG Results

Considering the alpha band, the ANOVA model for repeated measures revealed a significant effect of the factor Phase (F (1, 29) = 33.11, *p* < 0.01, η^2^ = 0.09), indicating a difference between the pre and post test results. This result is confirmed by the results in each single hemisphere in Figure 1 and Table 2. The Group X Phase interaction showed significant effects (F (1, 59) = 40.61, *p* < 0.01, η^2^ = 0.11. To further explore the nuances of the Group X Phase interaction, contrast terms were applied to investigate where precisely the effect occurred and its directional impact. Specifically, contrasts were utilized to assess the significance of differences between pre-test and post-test within both the experimental and control groups. Results indicated that contrast *C*_1_, representing the difference in the alpha band between pre-test and post-test values for the experimental group (*C*_1_ = *μ*Exp, Post − *μ*Exp, Pre), was statistically significant (*p* < 0.05). Similarly, contrast *C*_2_, representing the corresponding difference for the control group *C*_2_ = *μ*Ctrl, Post – *μ*Ctrl, Pre), was not significant (*p* = 0.11). These contrast terms provide a more detailed understanding of how the “Group X Phase” interaction manifests in the alpha band. Moreover, nor hemisphere side, neither phases X hemisphere side, neither group X hemisphere side showed significant effects. Considering the beta band, a significant effect of the factor Phase emerged (F (1, 29) = 5.27, *p* < 0.01, η^2^ = 0.11), indicating a difference between the pre and post test results. The Group X Phase interaction showed also significant effects (F (1, 59) = 35.73, *p* < 0.01, η^2^ = 0.11, suggesting that neurocognitive stimulation using Erigo device resulted in significant differences between the EG and CG in the beta frequency bands. Again, contrasts were utilized to assess the significance of differences between pre-test and post-test within both the experimental and control groups. Results indicated that contrast *C*_1_, representing the difference in the beta band between pre-test and post-test values for the experimental group (*C*_1_ = *μ*Exp, Post − *μ*Exp, Pre), was statistically significant (*p* < 0.05). Similarly, contrast *C*_2_, representing the corresponding difference for the control group *C*_2_ = *μ*Ctrl, Post – *μ*Ctrl, Pre), was not significant (*p* = 0.24). These contrast terms provide a more detailed understanding of how the “Group X Phase” interaction manifests in the alpha band. Again, neither hemisphere side, neither phases X hemisphere side, neither group X hemisphere side showed significant effects. Considering the theta band, no difference between the pre and post test results emerged. Nor hemisphere side neither phases X hemisphere side shows significant effects.

**Figure 2:**
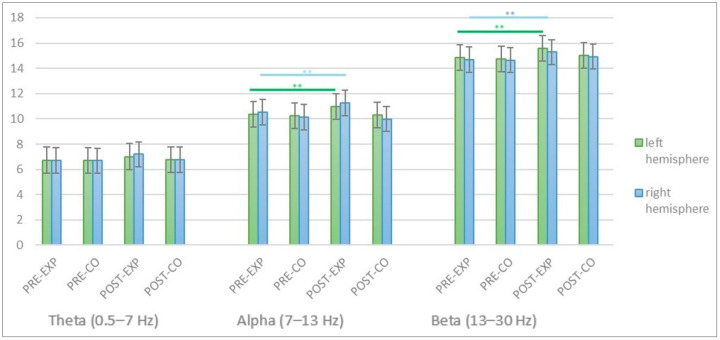
The bar plot shows the EEG bands power trend at pre- and at post- treatment for both groups. Legend: PRE-EXP (pre-treatment-experimental group), PRE-CO (pre-treatment-control group), POST-EXP (post-treatment-experimental group), POST-CO (post-treatment-control group).

In Figure 2, we reported a visual representation of the statistical comparisons, highlighting the observed differences in clinical outcomes between groups and over time. Specifically, each subplot corresponds to a different clinical measure and comparison. The *x*-axis represents the time points (pre and post), while the *y*-axis displays the clinical measure values. The colored bars or lines indicate the mean values for each group or condition, and error bars provide insights into the variability of the data. Significant differences are denoted by asterisks (**) and horizontal brackets connecting the groups or conditions with statistically significant contrasts.

Summarizing, the ANOVA model, applied to the EEG data, revealed significant effects for the alpha and beta bands. Specifically, in the alpha and beta bands, a significant Phase effect indicated differences between pre and post-test results. The Group X Phase interactions further emphasized the distinction between the experimental and control groups. Contrasts were employed to delve deeper into this interaction, with *C*_1_ showing a significant difference in the alpha band for the experimental group, while *C*_2_ revealed no significant difference for the control group.

Conversely, the theta band exhibited no significant differences in either Phase or Group X Phase interaction. Hemisphere side, Phase X Hemisphere side, and Group X Hemisphere side did not yield significant effects across all frequency bands.

### 3.2. Clinical Results

Regarding the LCF, the repeated measures ANOVA model revealed a significant effect of the Phase factor (F (1, 29) = 18.11, *p* < 0.01, η^2^ = 0.09), signifying a distinction between pre and post-test results. The Group X Phase interaction also demonstrated significant effects (F (1, 28) = 3.45, *p* < 0.05, η^2^ = 0.11). Moreover, the significant Group X Phase interaction implies that the observed difference is influenced by the type of intervention (RVT vs. VT), highlighting the specific impact of each intervention on cognitive functioning in post-coma patients. To further explore these effects, contrasts were applied to examine the significance of differences between pre-test and post-test within both the experimental and control groups. Results indicated that Contrast *C*_1_, representing the difference in LCF scores between pre-test and post-test values for the experimental group (*C*_1_ = *μ*Exp, Post − *μ*Exp, Pre), was statistically significant (*p* < 0.05). Conversely, Contrast *C*_2_, representing the corresponding difference for the control group (*C*_2_ = *μ*Ctrl, Post − *μ*Ctrl, Pre), did not reach statistical significance (*p* = 0.31). These findings suggest an improvement in cognitive functioning over the intervention period, with the type of intervention playing a significant role, as indicated by the significant Group X Phase interaction.

Considering the FIM, a significant effect of the factor Phase emerged (F (1, 28) = 25.47, *p* < 0.01, η^2^ = 0.09), indicating a difference between the pre and post test results. The Group X Phase interaction also showed significant effects (F (1, 28) = 10.56, *p* < 0.01, η^2^ = 0.10. The significant effect of the factor Phase in the ANOVA model suggests a notable increase in overall FIM scores between the pre and post-test assessments. Contrasts were utilized to assess the significance of differences between pre-test and post-test within both the experimental and control groups. Results indicated that contrast *C*_1_, representing the difference of FIM scores between pre-test and post-test values for the experimental group (*C*_1_ = *μ*Exp, Post − *μ*Exp, Pre), was statistically significant (*p* < 0.05). Similarly, contrast *C*_2_, representing the corresponding difference for the control group *C*_2_ = *μ*Ctrl, Post − *μ*Ctrl, Pre), was not significant (*p* = 0.17). This signifies an improvement in the participants’ physical, psychological, and social functions over the course of the intervention, regardless of the type of intervention. Moreover, the significant Group X Phase interaction implies that the observed improvement in FIM scores is influenced by the type of intervention (RVT vs. VT). This emphasizes that the increase in functional independence is not uniform across groups and indicates that the Erigo-assisted rehabilitation may contribute uniquely to the enhancement of overall functional independence in comparison to the control group.

Furthermore, to provide a comprehensive assessment, we applied paired *t*-tests within each group (CG T0–T1 and EG T0–T1) and independent *t*-tests between the two groups (CG vs. EG at T0 and at T1) for clinical outcome measures such as LCF and FIM. We identified statistical significance only in the EG between T0 and T1, with respect to LCF (*p* < 0.01) and FIM (*p* < 0.01), as illustrated in Table 3.

## 4. Discussion

The present study sought to investigate the impact of RVT using the Erigo device on various brainwave parameters, specifically alpha, beta, and theta bands in both left and right hemispheres, after PSD calculation. Our findings reveal significant changes in alpha and beta bands post-intervention, underscoring the promising effect of the Erigo device to influence neural plasticity. This effect in the alpha and beta bands was not observed in the CG. The increase in alpha power suggests a modulation of the brain’s oscillatory activity, possibly linked to enhanced cognitive processes or improved attention [29]. This finding aligns with the previous literature data emphasizing the role of motor activity and sensory stimulation in influencing neural oscillations [30,31,32,33]. Similarly, the beta band exhibited a significant effect, suggesting a distinct impact of the Erigo device on this frequency range. The heightened beta power post-intervention could be indicative of increased alertness or engagement, corroborating the notion that motor stimulation contributes to changes in cortical excitability and arousal level [34]. These findings support the hypothesis that the Erigo device, with its motor rehabilitation and sensory stimulation capabilities, contributes to distinct alterations in brainwave patterns associated with neural plasticity. Conversely, no significant differences emerged in the theta band, indicating stability in this frequency range following RVT, using the Erigo device.

Studies investigating the effects of robotic verticalization, and sensory-motor stimulation have reported similar findings, suggesting a positive impact on brain dynamics, particularly in the alpha frequency range [2,3,35]. Similarly, the significant effect in the beta band aligns with the literature emphasizing the role of motor stimulation in altering cortical excitability and arousal levels [4]. Robotic-assisted therapies have been shown to contribute to increased alertness and engagement, as reflected in changes in beta power [5,6]. According to Yue et al. [36], significant beta power variations at pre- and post-robot-assisted are positively correlated with intervention-induced motor improvements. Previous studies have shown that beta oscillation plays a role in motor learning [37,38,39], and it appears to be associated with increased gamma-aminobutyric acid (GABA) levels [40,41,42], suggesting its role in promoting plasticity [38,39]. A previous study by our group showed that RVT with Erigo in chronic stroke patients improved postural changes and lower limb functions, thanks to standardized, intensive, and repetitive exercises, with an appropriate sensory feedback amount and controlled progressive verticalization [43]. These positive results could be related to the fact that Erigo can provide unique afferent stimulation intensively and safely, especially, but not only, in a very early stage of rehabilitation. The robotic leg movement and the cyclic leg loading offered by the device are critical afferent stimuli for the central nervous system. Moreover, verticalization may also improve brain and CSF hemodynamics. Indeed, the device could also improve muscle activation, through a better muscle pump function, and venous return ultimately resulting in improved cardiovascular stability and brain circulation [18,44,45,46].

All of these improvements may lead to better neural activity and neural plasticity, as somehow demonstrated by the EEG analysis, and eventually to a more evident cognitive and functional recovery. In this regard, Taveggia et al. [47] investigated the role of RVT with Erigo to reduce orthostatic hypotension in patients with VS. They found that heart rate can be stabilized better by treatment with passive leg movements in hemodynamically unstable patients. A recent systematic review [13] suggested using Erigo to meet the needs of patients with post-severe traumatic brain injury or with DoC. In particular, the authors suggest its use to achieve an early and gradual verticalization to avoid deterioration of the autonomic nervous system and bedridden complications. Afterward, the gradual recovery in hemodynamic stability could allow robotic-assisted gait training with other devices, like Lokomat (Hocoma, Switzerland), which can assist a passive gait increasing BWS, always monitoring vital parameters to guarantee a safe and feasible rehabilitative intervention and to further promote motor recovery [48]. Moreover, no notable difference or impact observed between the brain hemispheres, or their interactions were found. This suggests that the device’s impact on brainwave modulation is not lateralized, supporting its potential as a versatile tool in motor rehabilitation.

Regarding clinical assessments, both LCF and FIM demonstrated significant differences between pre and post-test measurements. The Group X Phase interaction emphasized that these differences were influenced by the type of intervention (Erigo vs. Control), highlighting the specific impact of each intervention on cognitive functioning and overall functional independence in MCS patients. These results are in line with our previous study, in which we found that RVT alone or plus music stimulation [15] improved the overall cognitive functioning, tested with LCF. Frazzitta et al. (2016) [46] found that patients with DoC improved their global functioning tested with Coma recovery scale (CSR-r) after RVT with Erigo, although scores in LCF at post-treatment were not significant. It was an authors’ opinion that LCF is less sensible to detect changes in comatose patients. The literature on hemispheric specificity in response to motor interventions varies, with some studies emphasizing lateralized effects and others highlighting non-lateralized changes in brain activity [9,10,34]. This stability might suggest that the device’s effects are more prominent in higher-frequency oscillations, reflecting specific cognitive processes related to attention and alertness, rather than broader changes in neural synchronization.

Among the limitations related to our study, the retrospective nature of this work constitutes a bias itself and RCT should be fostered to confirm whether treatment with Erigo is more effective than conventional treatment. Secondly, a generalization of our results is impossible due to the small number of DoC patient subjects involved in our sample. Additionally, according to our opinion, LCF scale is not the best to rate the level of consciousness. For this reason, we consider its use as another study’s limitation and for futures studies, we would like to use more elegant and sensitive tools, to measure consciousness, including also the Come Recovery Scale—revised (CRS-R). Moreover, we have administered the SECONDs scale only to T0 phase, to classify Docs’ patients in plus or minor, in in relation to the scores obtained for specific clinical condition. However, this use could be reductive, and we believed that it could be useful to administer it also to T1 phase, to evaluate also specific outcome. Finally, we did not investigate the long-term effects of Erigo, so we are not able to state if and to what extent the improvement in awareness and motor functions lasts.

## 5. Conclusions

In conclusion, our study provides empirical evidence supporting the hypothesis that exercise and motor stimulation, facilitated by the Erigo device, can modulate brain activity. The distinct alterations in alpha and beta bands signify the potential of Erigo as a valuable tool in promoting neural plasticity, thus opening avenues for further exploration and application in neurological rehabilitation. Further larger sample studies with long-term outcomes are needed to confirm these promising results and investigate whether and to which extent Erigo training after-effects may last.

## Figures and Tables

**Table 1:** Socio-demographic clinical description of the study sample.

Patients	EG N = 15	CG N = 15	All N = 30	*p*-Value
Age	58.13 ± 8.33	57.33 ± 11.06	57.73 ± 9.63	0.8
Educational level				
Elementary school	1 (6.66%)	3 (20.00%)	4 (13.33%)	0.56
Middle school	8 (53.33%)	7 (46.66%)	15 (50.00%)	
High school	6 (40.00%)	5 (33.33%)	11 (36.67%)	
Gender				
Male	10 (66.67%)	10 (66.67%)	20 (66.67%)	1
Female	5 (33.33%)	5 (33.33%)	10 (33.33%)	
Etiology				
Vascular	10 (66.67%)	10 (66.67%)	20 (66.67%)	1
Traumatic	5 (33.33%)	5 (33.33%)	10 (33.33%)	
SECONDs	3.73 ± 1.43	3.46 ± 1.76	3.6 ± 1.58	0.65
MCS+	3 (20.00%)	3 (20.00%)	6 (20.00%)	1
MCS−	12 (80.00%)	12 (80.00%)	24 (80.00%)	

Legend: SECONDs (Simplified Evaluation of CONsciousness Disorders). The designation of MCS+ pertains to patients exhibiting specific pivotal behaviors, including but not limited to consistent and reproducible responses to commands, recognition of objects, and intelligible verbalization with deliberate (albeit non-functional) communication. Conversely, MCS− denotes individuals characterized by manifestations such as reaching, visual pursuit, fixation, object manipulation, and automatic motor responses; EG (Experimental group), CG (Control group).

**Table 2:** Means and Standard Deviations of Theta, Alpha, and Beta Bands (Microvolts) in right and left hemispheres and *p*-values.

	Pre-Test		*p*	Post-Test		*p*
Measures	Experimental	Control		Experimental	Control	
Left hemisphere						0.14
Theta band	6.73 (0.43)	6.73 (0.55)	0.22	7.01 (0.42)	6.78 (0.53)	
Alpha band	10.36 (2.01)	10.26 (1.47)	0.36	10.99 (1.65)	10.22 (0.68)	0.05
Beta band	14.85 (2.11)	14.75 (1.62)	0.49	15.59 (1.86)	14.88 (1.40)	0.01
Right hemisphere						0.14
Theta band	6.72 (0.43)	6.69 (0.57)	0.22	7.20 (0.43)	6.77 (0.54)	
Alpha band	10.53 (1.40)	10.14 (1.36)	0.36	11.26 (0.87)	9.98 (0.78)	0.05
Beta band	14.67 (1.46)	14.65 (1.62)	0.49	15.48 (1.23)	14.93 (1.35)	0.01

**Table 3:** Paired and independent t test for clinical scales.

Clinical Measures		Mean (SD)	*p*-Value
LCF	CG T0–EG T0	3.13 (1.41)–3.60 (1.05)	0.31
	CG T0–CG T1	3.13 (1.41)–3.60 (1.50)	0.09
	EG T0–EG T1	3.60 (1.05)–4.40 (1.06)	<0.01 *
	CG T1–EG T1	3.60 (1.50)–4.40 (1.06)	0.1
FIM	CG T0–EG T0	20.53 (5.39)–20.27 (4.59)	0.49
	CG T0–CG T1	20.53 (5.39)–21.10 (1.62)	0.13
	EG T0–EG T1	20.27 (4.59)–23.40 (5.86)	<0.01 *
	CG T1–EG T1	21.10 (1.62)–23.40 (5.86)	0.36

Legend: EG (experimental group), CG (control group), LCF (level of cognitive functioning), FIM (functional independence measure); * *p*-values in bold are statistically significant; Statistical comparisons were obtained through *t*-test, with an alpha level of 0.05 and a Bonferroni correction of 0.0125.

## Data Availability

Data as well as MATLAB scripts will be available on demand, from the corresponding author. The data is not publicly available due to privacy reasons.
